# Combining release and runout in statistical landslide susceptibility modeling

**DOI:** 10.1007/s10346-019-01222-7

**Published:** 2019-07-01

**Authors:** Martin Mergili, Leonhard Schwarz, Arben Kociu

**Affiliations:** 1 Geomorphological Systems and Risk Research, Department of Geography and Regional Research, University of Vienna, Universitätsstraße 7, 1010 Vienna, Austria; 2 Institute of Applied Geology, University of Natural Resources and Life Sciences (BOKU), Peter-Jordan-Straße 82, 1190 Vienna, Austria; 3 Department of Engineering Geology, Geological Survey of Austria, Neulinggasse 38, 1030 Vienna, Austria

**Keywords:** GIS raster analysis, Landslide susceptibility, Landslide runout, Statistical model

## Abstract

We introduce and compare two approaches to consistently combine release and runout in GIS-based landslide susceptibility modeling. The computational experiments are conducted on data from the Schnepfau investigation area in western Austria, which include a high-quality landslide inventory and a landslide release susceptibility map. The two proposed methods use a constrained random walk approach for downslope routing of mass points and employ the probability density function (PDF) and the cumulative density function (CDF) of the angles of reach and the travel distances of the observed landslides. The bottom-up approach (A) produces a quantitative spatial probability at the cost of losing the signal of the release susceptibility, whereas the top-down approach (B) retains the signal and performs better, but results in a semi-quantitative score. Approach B also reproduces the observed impact area much better than a pure analysis of landslide release susceptibility. The levels of performance and conservativeness of the model results also strongly depend on the choice of the PDF and CDF (angle of reach, maximum travel distance, or a combination of both).

## Introduction

Overviews of spatial landslide probability (susceptibility) at local or regional scales are useful to support hazard indication zonation and for prioritizing target areas for risk mitigation. Computer models that utilize geographic information systems (GIS) are commonly employed to produce such overviews (Van Westen et al. 2006). With ever increasing computational power, physically based modeling of landslide susceptibility—also with reasonably complex modeling tools—is increasingly becoming not only technically feasible but also able to be conducted for large areas (Mergili et al. 2014a, b). However, slopes are often not uniform, but characterized by multi-scale patterns in terms of the rock or soil characteristics governing their stability (De Lima Neves Seefelder et al. 2017). Since physically based models require this type of information, the parameterization of these models becomes a highly challenging task, leading to limitations of the quality of the results obtained. Heuristic models, based on the opinion of experts, are useful for larger areas, but they often provide qualitative results only. For these reasons, statistical methods—often coupled with stochastic concepts—are commonly employed to relate the spatial patterns of landslide occurrence to those of environmental variables such as slope, vegetation, or lithology, and applying these relationships to estimate landslide susceptibility (Guzzetti 2006). A broad array of statistical methods for landslide susceptibility analysis has been developed, documented by a large number of publications (e.g., Carrara et al. 1991; Baeza and Corominas 2001; Dai et al. 2001; Lee and Min 2001; Brenning 2005; Saha et al. 2005; Guzzetti 2006; Komac 2006; Lee and Sambath 2006; Lee and Pradhan 2007; Yalcin 2008; Yilmaz 2009; Schwarz et al. 2009; Nandi and Shakoor 2010; Yalcin et al. 2011; Tilch et al. 2011; De Graff et al. 2012; Petschko et al. 2014; Schwarz and Tilch 2017). Such methods only consider the release of landslides and not their further movement down slopes or channels.

The propagation of landslides often contributes substantially to the associated hazards and risks: the most severe landslide disasters in history occurred far away from the release areas. The 1962 and 1970 Huascarán events in Peru (Evans et al. 2009; Mergili et al. 2018) are only two examples of catastrophic long-runout landslides. Even though most cases are less extreme, particularly those which initiate at steeper slopes tend to evolve into flow-like movements with potentially long runout distances even on more gentle slopes. Therefore, simulations of such mass flows are important to anticipate their consequences. Advanced physically based models of landslide propagation (Christen et al. 2010a, b; Mergili et al. 2017) are usually employed for local-scale studies. Conceptual approaches, in contrast, have been developed to analyze and to estimate travel distances and impact areas at broader scales. Some build upon the angle of reach or related parameters (e.g., Scheidegger 1973 for rock avalanches; Zimmermann et al. 1997 and Rickenmann 1999 for debris flows; Corominas et al. 2003 for various types of landslides; Noetzli et al. 2006 for rock/ice avalanches), and others consist of semi-deterministic models employing the concept of Voellmy (1955) (Perla et al. 1980; Gamma 2000; Wichmann and Becht 2005; Horton et al. 2013). Mergili et al. (2015) have introduced an automated approach to statistically derive cumulative density functions of the angle of reach from a given landslide inventory and to apply these functions to compute a spatially distributed impact probability. Modeling approaches considering both the release and the propagation of landslides do exist (Mergili et al. 2012 and Horton et al. 2013 for debris flows; Gruber and Mergili 2013 for various high-mountain processes). However, they yield expected impact or deposition depths, binary results (impact/no impact expected), or semi-quantitative scores.

Integrated automated quantitative approaches to properly estimate the susceptibility of a given area to be affected by a landslide—considering both release and propagation—are still scarce. We postulate that such approaches would be highly important to better anticipate those areas most likely impacted by future landslides, compared to approaches covering either of the process components. The outcome of such a model would therefore represent an extremely valuable basis for hazard and risk management, particularly with regard to spatial planning and to the prioritization of areas requiring further research.

The present work attempts to elaborate on this gap by combining the two open-source software tools r.landslides.statistics and r.randomwalk. The combination of these tools is much more than just a technical issue: instead, we focus on strategies for the appropriate spatial combination of the impact probabilities or susceptibility indices related to different release areas or cells. Thereby, we partly build on an earlier attempt of Mergili and Chu (2015). When using the term “landslide” in the context of the present work, we mostly refer to shallow landslides developing into hillslope debris flows.

We will next introduce our computational framework and the Schnepfau investigation area in western Austria along with the data employed and the computational experiments performed on this area. After presenting and discussing the results, we will conclude with a set of take-home messages.

## Methodical framework

### General model layout

We introduce a statistical-stochastic framework to compute the spatial pattern of the likelihood of landslide impact within a given hilly or mountainous landscape, which is subdivided into a regular grid of GIS raster cells. For this purpose, we use an existing map provided by the Geological Survey of Austria (Schwarz and Tilch 2018), depicting the spatial patterns of the likelihood of landslide initiation (thereinafter referred to as landslide release susceptibility index *LRSI*). All probabilities, susceptibilities, and their abbreviations used in the present work are summarized in Table 1. Onto this map, we impose a landslide propagation model routing the unstable material downslope through the landscape, represented by a digital terrain model (DTM). A constrained random walk approach is used for routing. Random walks are a type of Monte Carlo simulation where points of mass are released from a given raster cell for several times, and each time take a different path downslope. This serves for realistically simulating the lateral spread of a landslide, opposed to a concentration along the steepest descent (which would be suitable for stream flow).

**Table 1:** Definition of the susceptibilities and probabilities used in the context of the present work

Variable	Approach	Name	Description	Type, range
*P*_R_	A	Landslide release probability	Spatial probability of a raster cell to become a landslide release cell.	Float, 0–1
*P*_IS_	A	Specific impact probability	Spatial probability of a raster cell to be impacted by the propagation of a mass point starting from one specific point.	Float, 0–1
*P*_I_	A	Impact probability	Spatial probability of a raster cell to be impacted by the propagation of mass points starting in its upslope contributing area.	Float, 0–1
*P*_RZ_	A	Zonal release probability	Spatial probability that landslide release occurs at all (from at least one raster cell) in the upslope contributing area of a given cell.	Float, 0–1
*P*_L_	A	Integrated landslide probability	Spatial probability that a given raster cell is directly affected by at least one landslide either through release or through propagation.	Float, 0–1
*LRSI*	B	Landslide release susceptibility index	Index denoting the likelihood of a raster cell to become a landslide release cell.	Float, 0–1
*ILSS*	B	Integrated landslide susceptibility score	Semi-quantitative score indicating the likelihood of a given raster cell to be directly affected by at least one landslide either through release or through propagation.	Integer, ≥ 0
*ILSI*	B	Integrated landslide susceptibility index	Semi-quantitative index derived by the normalization of *ILSS* to the range 0–1.	Float, 0–1

We compare two different approaches for combining landslide release and propagation:

A. Bottom-up approach: for each raster cell (“impact cell”), the probability that landslide release is observed anywhere in its catchment is computed. This zonal release probability *P*_RZ_ is calculated from the landslide release susceptibility index at each of the GIS raster cells in the catchment of the impact cell in combination with the size of the catchment. *P*_RZ_ is then multiplied with the probability that the same impact cell is reached by a landslide released in its catchment (impact probability *P*_I_) in order to derive the integrated landslide probability *P*_L_. *P*_I_ is derived from the cumulative density function of the angles of reach and the travel distances of the past observed landslides in the study area.
B. Top-down approach: a set of random walks proportional to the landslide susceptibility index is started from each raster cell (“release cell”). Each random walk proceeds downslope until a threshold angle of reach or threshold travel distance is met. Individual thresholds for each random walk are probabilistically deduced from the angles of reach and the travel distances of the observed landslides in the study area. Each time a raster cell is impacted by a random walk, its integrated landslide susceptibility score *ILSS* is increased by 1.

**Fig. 1:**
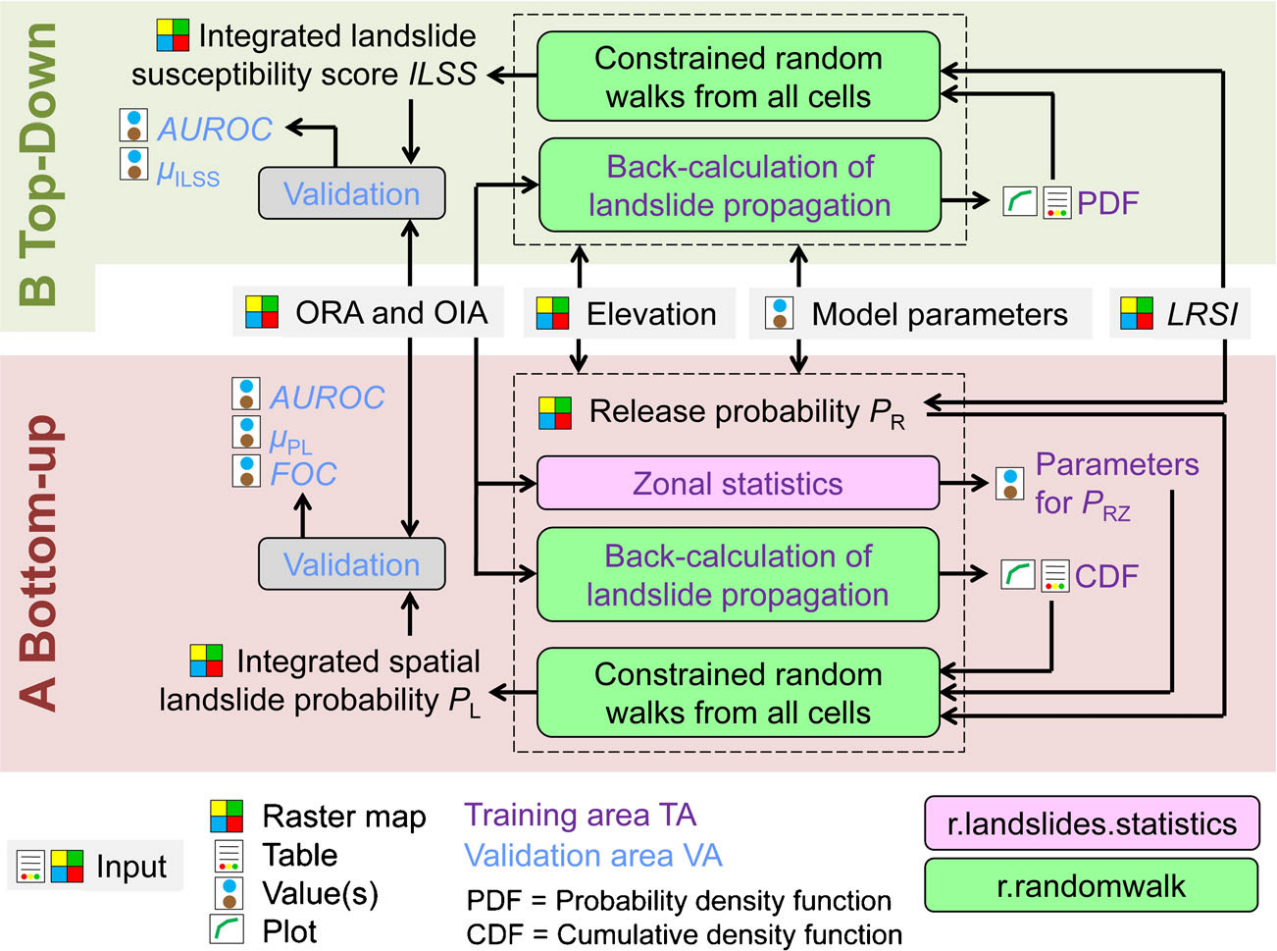
Simplified work flow of the integrated statistical analysis of landslide susceptibility. Elevation, ORA, and OIA maps as well as a *LRSI* map, and a set of model parameters are the main input for both approaches A and B

The entire work flow is illustrated in Fig. 1. Approach B is simpler and more intuitive, but results in a semi-quantitative score, whereas approach A produces a real spatial probability in the range 0–1: in the ideal case, the average of the integrated landslide probability is roughly identical to the observed landslide density. Besides the DTM, both approaches rely on a landslide inventory and on an *LRSI* map. The inventory has to differentiate between the observed landslide release areas (ORA) and the observed transit and deposition areas. The release, transit, and deposition areas together form the observed impact area of a given landslide (OIA) (Table 2).

**Table 2:** Abbreviations used to describe the spatial subsets of the investigation area

Abbreviation	Name	Description
TA	Training area	Subset of all raster cells within the study area used for the derivation of zonal statistics, CDFs, and PDFs
VA	Validation area	Subset of all raster cells within the study area used for model validation
OIA	Observed landslide impact area	Subset of all raster cells within the study area with observed landslides (including release, transit, and deposition areas)
ORA	Observed landslide release area	Subset of all raster cells within the study area with observed landslide release

Two tool sets implemented as raster modules of the open-source software package GRASS GIS 7 (Neteler and Mitasova 2007; GRASS Development Team 2018) are combined for the analyses:

- r.landslides.statistics has been designed for the generation of the zonal probability function needed as part of the approach A.
- r.randomwalk, introduced by Mergili et al. (2015), employs sets of constrained random walks to route hypothetic mass points—representing landslides—down through a DTM until a certain break criterion is met (approach B), optionally assigning an impact probability to each raster cell it hits (approach A). The probability density function (PDF) and the cumulative density function (CDF) used for this purpose are derived from the analysis of the observed landslides. Further, r.randomwalk includes an algorithm to combine release and impact probability, employing the zonal release probability function derived with r.landslides.statistics (approach A).

### Approach A (bottom-up): integrated landslide probability

#### Release susceptibility and probability

Statistical analyses of landslide release susceptibility have been treated exhaustively in previous studies (see “Introduction” for selected references) and are not the focus of the present work. Therefore, we use an existing map of the release susceptibility index *LRSI* (see Schwarz and Tilch 2018 for details). *LRSI* is an index within the range 0–1, where higher values stand for a higher susceptibility to landslide release, and lower values stand for a lower susceptibility to landslide release. For approach A, we have to convert the *LRSI* map into a spatial release probability *P*_R_ map. In contrast to *LRSI*, *P*_R_ has to be in line with the spatial probability of observed landslide release areas. Its average over the entire study area has to be identical to the “density” of observed landslides. For example, if, in a study area of 10 km^2^, 0.1 km^2^ are classified as observed landslide areas, this would correspond to a “landslide density” of 0.01 (1%). The *P*_R_ map for the same area would have to show an average of 0.01 over all GIS raster cells, in order to be consistent with the observation.

In a case where the values of *LRSI* shown in the release susceptibility map are proportional to the spatial release probabilities, the map of *P*_R_ can be derived by linear scaling of the values of *LRSI*.

#### Zonal release probability

It is useful in many contexts to work with GIS raster cell-based spatial release probabilities *P*_R_. They can be averaged in order to characterize the likelihood of landsliding in any type of landscape unit (such as slope units, catchment basins, or cells resampled to a coarser resolution). However, the average of *P*_R_ over a given landscape unit does not tell us how probable it is that landsliding occurs in that zone at all. Therefore, we take up the concept of the zonal release probability *P*_RZ_ first suggested by Mergili and Chu (2015), which increases with the size *Z* of the considered zone. *P*_RZ_, which can take values in the range 0–1, represents the probability that at least one landslide initiates in a given zone and is based on the observed patterns of landslide release areas. When considering a zone size of one single GIS raster cell, *P*_RZ_ = *P*_R_. For large areas such as mountainous catchments, *P*_RZ_ = 1 as there will always be at least one observed landslide release cell. We emphasize that *P*_RZ_ is always related to a zone of a given size rather than to a raster cell. In the present work, *P*_RZ_ assigned to each cell relates to its upslope contributing area, which is almost similar to its hydrological catchment area. This concept is needed for approach A as a basis to compute the integrated spatial landslide probability *P*_L_. *P*_RZ_ cannot be computed in a fully analytic way. Consequently, we introduce an empirical approximation procedure described in detail in the Appendix.

#### CDFs of break criteria for landslide propagation

**Fig. 2:**
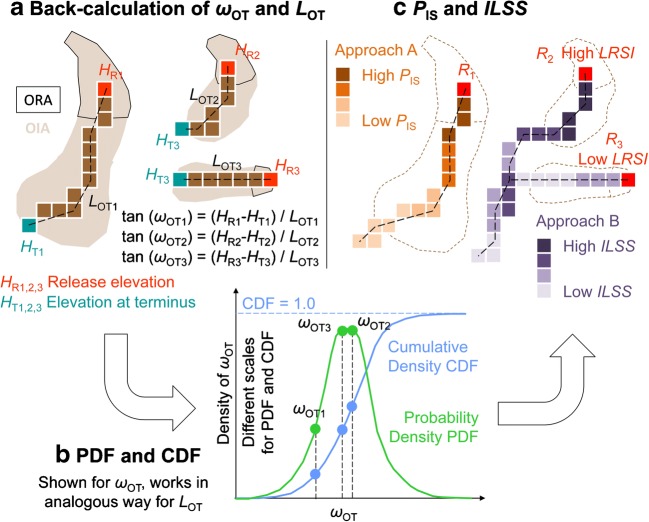
Work flow for estimating the impact probability *P*_I_ (approach A) and the integrated landslide susceptibility score *ILSS* (approach B) with the tool r.randomwalk. (a) Back-calculation of observed values of the angle of reach *ω*_OT_ and the observed travel distance *L*_OT_. One set of random walks starts from each cell of the ORAs (represented by the areas enclosed by the dark solid lines) of all observed landslides. Shaded areas represent the OIAs. All random walks stop when leaving the corresponding OIA, and *ω*_OT_ and *L*_OT_ are recorded. For clarity, only one random walk is shown for each of three selected release cells. (b) PDF and CDF derived for *ω*_OT_, based on the values derived in (a) for all release cells. (c) Computation of *P*_IS_ and *ILSS*, exemplified with the same release cells as in (a) and based on the CDF (approach A) or PDF (approach B) derived in (b). Note that *P*_IS_ is illustrated for the release cell *R*_1_, whereas *ILSS* is shown for the release cells *R*_2_ and *R*_3_. Only one random walk is shown for the release cell *R*_1_, whereas for the release cells *R*_2_ and *R*_3_, it is assumed that all random walks take the same path. In reality, r.randomwalk starts sets of random walks from all cells within the study area, and all random walks will take slightly different paths

The computational tool r.randomwalk (Mergili et al. 2015) is applied for analyzing the angle of reach (overall slope measured along the flow path) and the travel distance of each observed landslide. Thereby, sets of constrained random walks are routed from each cell of the ORA of each observed landslide down through the DTM. Each random walk stops as soon as it reaches the point where it leaves the OIA of the same landslide. The observed angle of reach *ω*_OT_ (Heim 1932) and the observed travel distance *L*_OT_ (measured along the ground plot of the flow path) are recorded for each landslide (Fig. 2(a)). Within the context of the present work, we consider these variables as most appropriate for parameterizing landslide mobility in a simple way.

The values of *ω*_OT_ and *L*_OT_ are collected for all observed landslides and are employed to build CDFs for each of the two criteria (Fig. 2(b)). They describe the probability that a landslide has not yet stopped when a certain threshold of *ω*_OT_ or *L*_OT_ has been reached.

#### Impact probability

The tool r.randomwalk is applied for this step. Thereby, a set of constrained random walks is started from each raster cell and routed down through the terrain until it reaches the boundary of the investigation area. A specific impact probability *P*_IS_ is assigned to each cell impacted by a random walk. *P*_IS_ describes the probability of an arbitrary GIS raster cell (“impact cell”) to be hit by a mass point released from a defined cell (“release cell”). We define *P*_IS_ based on the angle of the path *ω* between the release cell and the impact cell, the distance along the same path *L*, or a combination of both. Thereby, the relevant CDFs are employed.

The impact probability *P*_I_ results from the spatial overlay of all relevant values of *P*_IS_ at a given cell (Table 1, Fig. 2(c)). For reasons to be explained in the following section on the integrated landslide probability, for those cells with impacts from more than one release cell, *P*_I_ takes the average value of all relevant values of *P*_IS_.

#### Integrated landslide probability

**Fig. 3:**
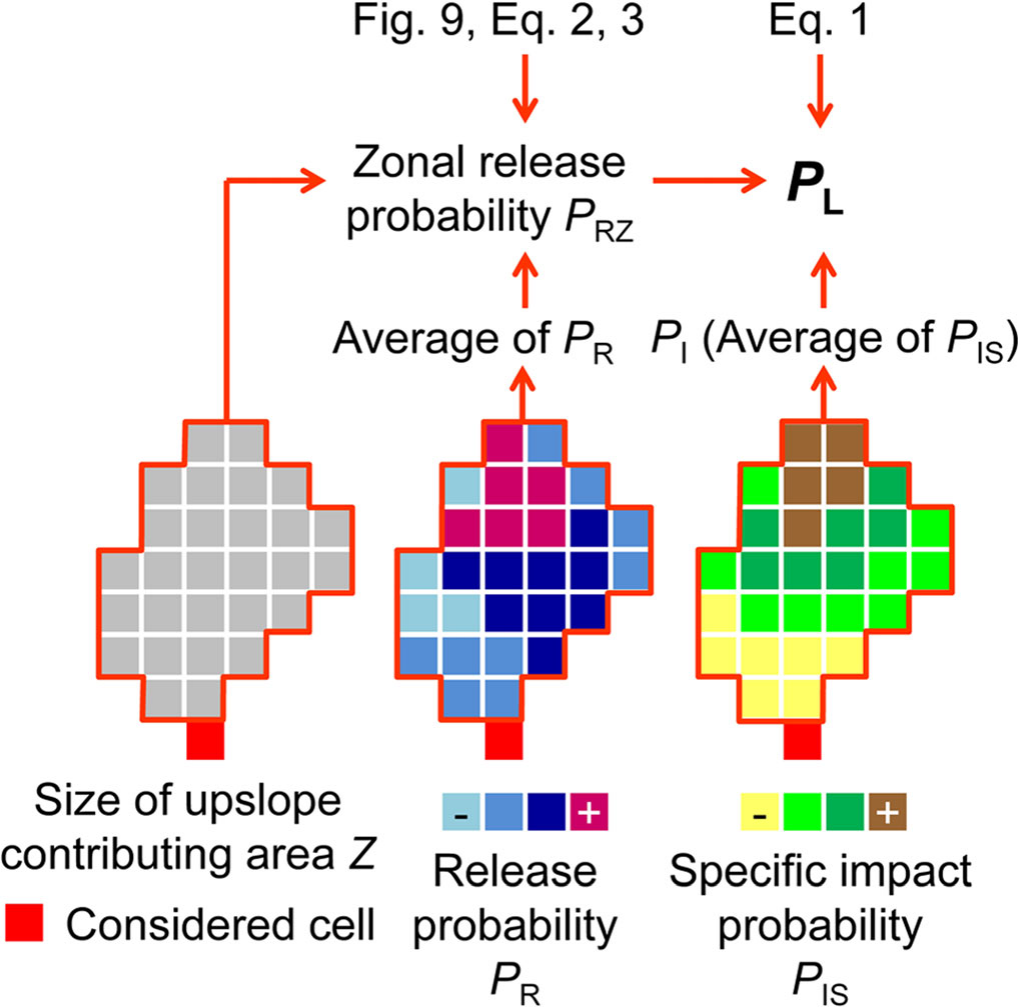
Integrated landslide probability *P*_L_ for a given GIS raster cell as a function of the release probability *P*_R_, the impact probability *P*_I_, and the size of the upslope contributing area *Z*. The average of *P*_R_ and the value of *Z* associated with the cell under consideration are employed along with Eq. 3 to compute the zonal release probability *P*_RZ_ (Fig. 2(c)). *P*_RZ_ and *P*_I_ are multiplied to compute *P*_L_ (Eq. 1). Note that, for readability, the values of *P*_IS_ are shown for the associated release cells even though they apply to the cell under consideration

The integrated landslide probability *P*_L_ approximates the probability that a landslide directly affects a given GIS raster cell (“impact cell”), either through its release or through its runout (Table 1). In principle, *P*_L_ is computed by multiplying a release probability and an impact probability. However, a simple overlay of *P*_R_ and *P*_I_ would be meaningless in this case. Instead, we have to consider for each impact cell with *P*_I_ > 0 the zonal release probability *P*_RZ_ of the upslope contributing area, roughly corresponding with the drainage basin, relevant for this cell. *Z* and the associated value of *μ*_PR_ (Fig. 3) refer to the upslope contributing area based on the outcome of the random walk routing procedure. The upslope contributing area includes the release areas of all hypothetic landslides impacting the considered cell. *P*_RZ_ has to be computed separately for each impact cell according to Eq. 3 (Appendix). *P*_L_ is then set to the product of *P*_RZ_ and *P*_I_:

1 $$ {P}_{\mathrm{L}}={P}_{\mathrm{RZ}}\cdot {P}_{\mathrm{I}} $$

For illustration, let us consider a given impact cell characterized by a value of *P*_RZ_ = 0.5 for its upslope contributing area, depending on *Z* and on the separate values of *P*_R_ for each “release cell” (all cells in the upslope contributing area are considered as release cells). Let us further assume that the upslope contributing area consists of two release cells only, so that the impact cell is characterized by two values of *P*_IS_ (0.8 and 0.2), each relating to a possible landslide impact from one of the two release cells in the upslope contributing area (see description of the impact probability). Working with the concept of the zonal release probability, (i) we assume that the release of landslides is equally probable for each release cell in the upslope contributing area, whereas (ii) the probability that landsliding occurs in this area at all is given by *P*_RZ_. It is important to note that *P*_RZ_ does not apply to each of the two release cells but represents an aggregated value also including the possibility of a release from both cells, or from none of the two cells. This means that the probability that a landslide reaches our impact cell can be approximated by the product of *P*_RZ_ and the average of *P*_IS_. In our example, this results in *P*_L_ = 0.25.

We note that the described procedure is supposed to yield smoothed results due to averaging effects: on the one hand, Eq. 3 builds on the simplification of a uniformly distributed release probability over the possible release zone. On the other hand, as highlighted in the section on the impact probability, *P*_I_ represents the average of *P*_IS_ over all mass points impacting a cell. This type of averaging is necessary to ensure a consistent combination of *P*_RZ_ and *P*_I_. We further note that also those cells with *P*_R_ = 0 are included as release cells. This means that *Z* and therefore also *P*_RZ_ are computed including those cells with *P*_R_ = 0. An alternative approach would consist in excluding those cells with *P*_R_ = 0 both from *Z* and from *P*_I_. Preliminary tests, however, have shown that this would not change the results in a substantial way.

### Approach B (top-down): integrated landslide susceptibility score

The integrated landslide susceptibility score *ILSS* (approach B) represents a more straightforward and intuitive, though semi-quantitative alternative to *P*_L_ (approach A). The release susceptibility map is used as the basis for the procedure. It can be directly used as input, without the pre-processing required for approach A.

The entire procedure is performed with the tool r.randomwalk. In a way analogous to approach A, the values of *ω*_OT_ and *L*_OT_ are collected for all observed landslides. They are employed to build probability density functions (PDFs) instead of CDFs for each of the two criteria (Fig. 2(b)). They describe the probability that a landslide stops at a certain threshold of *ω*_OT_ or *L*_OT_. A set of constrained random walks is started from each raster cell with *LRSI* > 0. Thereby, the number of random walks *n*_W_ is proportional to *LRSI*: *n*_W_ = int(100· *LRSI* + 0.5), meaning that 100 random walks start from cells with *LRSI* = 1.0, whereas 10 random walks start from cells with *LRSI* = 0.1, etc. The break criterion for each single random walk is randomly set according to the PDF of the angle of reach, the travel distance, or a combination of both. This means that more random walks use the more probable values of the break criteria, whereas fewer random walks use the less probable values.

Each time a random walk hits a raster cell (including the release cell), the landslide susceptibility score *ILSS* for this cell is increased by 1. At the end, those cells which are likely to be reached by landslides released from cells with high values of *LRSI* display high values of *ILSS*, whereas those cells less likely to be impacted by landslides released from cells with low values of *LRSI* show low values of *ILSS* (Fig. 2(c)). In order to facilitate the validation of the results by means of ROC plots, *ILSS* is converted to the index *ILSI* through normalization of its values to the range 0–1.

## Investigation area and model parameterization

### The Schnepfau investigation area

The Schnepfau investigation area covers an area of 58 km^2^ and is situated in the Bregenzerwald which is part of the federal state of Vorarlberg, western Austria. The area is located on the east bank of the Bregenzerach River and comprises parts of flat alluvial plains, mostly low mountain ranges, and partly also high mountains up to 1834 m. Hence, the morphology of the area is ranging from flat and hilly to steep and rocky parts.

The investigation area is mostly composed of geological units of the Säntisdecke as part of the Helvetikum (Friebe 2007), consisting of a variety of rocks such as marl, sandstone, and limestone. The formations of the Säntisdecke generally show northwards overturned folding, whereby the fold axes indicate a stretching in WSW–ENE direction (Oberhauser 1951). Intersection with the surface can result in large, homogeneous or smaller, heterogeneous outcrops. Generally, large, homogeneous areas are found in the southern (Palfris Formation, Quintner Limestone) and central parts (Schratten Limestone) of the investigation area, whereas small heterogeneous areas exist in the central (e.g., Palfris Formation) and northern parts (e.g., Schratten Limestone, Drusberg Formation).

The competent, often karstified and bare carbonate rocks of Quintner Limestone and Schratten Limestone and their weathering products are the most widespread examples for geological formations hardly susceptible to shallow landslides and hillslope debris flows. In contrast, rocks such as the marl slates of the Palfris Formation and the marls of the Drusberg Formation weather to clay-rich material highly susceptible to shallow landslides. Moreover, the Palfris and Drusberg Formations often show shallow embedded limestone layers inside the marls (Oberhauser et al. 1991), resulting in quite a heterogeneous landslide susceptibility. Further, wide areas are covered by glacial till (mostly on south-facing slopes) or debris, whereby both materials can be quite susceptible to landslides, depending on their often allochthonous or parautochthonous origin. Formations with an intermediate level of landslide susceptibility are less common in the investigation area.

### Input data

For the runout map, we use a 5 × 5 m resolution DTM, deduced and resampled from an aerial LiDAR point cloud and provided by the Federal Government of Vorarlberg (Landesamt für Vermessung und Geoinformation). This DTM represents the situation before the occurrence of most landslides shown in the inventory. Those raster cells coinciding with stream lines at the bottom of valleys are set to no data, so that the runout simulations stop there and those cells are excluded from all analyses. This kind of pre-processing is necessary as the model approaches chosen and the data available do not support the analysis of landslides directly connected to the valley bottoms or even interacting with streams (such as channelized debris flows or river erosion) (see “Discussion”).

The landslide inventory includes mainly shallow landslides, triggered by several extreme meteorological events. Many of the initial landslides have evolved into hillslope debris flows. Eight series of aerial photos (1950 to 2012) and one LiDAR DTM were available and used for interpretation. River erosion and uncertain landslide data had to be excluded from the dataset. In this way, it was possible to create a high-quality and reasonably complete landslide inventory. Two hundred forty-two hillslope debris flows were delineated as polygons, serving as input for runout modeling. Separate polygons are available for (i) the release areas (ORA) and (ii) the transit and deposition areas of the observed landslides even though, for some landslides, it is not possible to clearly assign a particular ORA to a given transit and deposition area, or vice versa. Further several transit and deposit areas do not show a clear end, because they are suddenly cut by a stream or their toe is covered by forest. All such landslides are excluded from the derivation of the PDFs and CDFs, but included in the validation. The key characteristics of the landslides in the inventory employed for parameterizing the runout simulations (*n* = 110) are summarized in Table 3.

**Table 3:** Key characteristics of the landslides observed in the Schnepfau investigation area

Parameter	ORA (*n* = 110)			OIA (*n* = 113)		
	Minimum	Average	Maximum	Minimum	Average	Maximum
Area (m^2^)	50	305	2450	100	1250	10,100
Projected length (m)	0	24	101	0	83	336
Average inclination (degree)	5.2	27.9	40.4	6.7	23.9	39.2

**Fig. 4:**
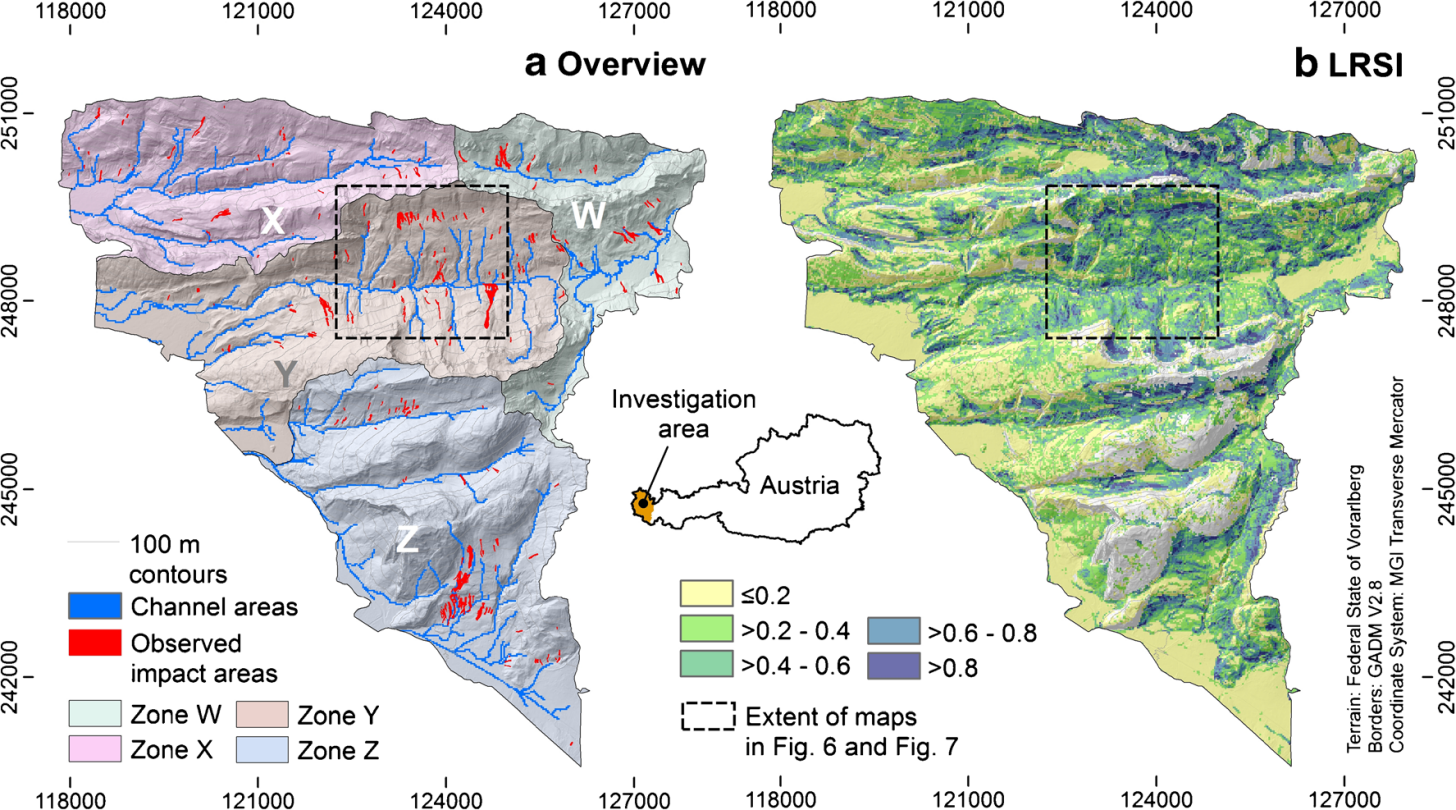
The Schnepfau investigation area in Vorarlberg, western Austria. (a) General overview with terrain and landslide inventory. W–Z refer to the subsets of the area alternatively used as VA (Table 3). (b) Map of the landslide release susceptibility index *LRSI* (Schwarz and Tilch 2018)

The applied, yet unpublished landslide release susceptibility map was produced by the Geological Survey of Austria, Department of Engineering Geology, using logistic regression (Fig. 4(b)). For the generation of several of its input variables, the same DTM as for the runout simulations was used, but resampled to 25 × 25 m. The landslide release susceptibility map is based on points, located in the release area of shallow landslides and hillslope debris flows. The validation of this map against the ORA-polygons, which were not used for its generation, results in *AUROC* = 0.796, indicating a fair to good performance. An even slightly better result (*AUROC* = 0.822) is achieved when validating the map against the test data of the landslide points, used for modeling this map (for details see Schwarz and Tilch 2018).

### Computational experiments

The six computational experiments performed within the present study are summarized in Table 4. The set of experiments is primarily designed to explore the difference in output and performance (i) between the approaches A and B, and (ii) between the various criteria applied to the random walks; and (iii) to learn about the degree to which the signal of *P*_R_ and *LRSI* “survives” the routing procedure and is represented in the final outcome. In this sense, three experiments 1–3 are conducted for each of the approaches A and B. Thereby, the criterion governing the random walks is set to the angle of reach (1), the projected travel distance along the flow path (2), and a combination of both (3), whereas all other parameters are kept constant. Within each experiment, a spatially uniform value of *P*_R_ (approach A) or *LRSI* (approach B), corresponding to its average over the investigation area, is considered alternatively to the actual map. This allows us to evaluate the signal of the release component in our results. For comparison, we also validate the *LRSI* map against the observed impact area (OIA). We are well aware that the meaning of such a validation is limited in terms of the process sub-areas involved, since the *LRSI* does not target the transit and deposition areas, and was therefore derived from the ORA and not from the OIA. Nevertheless, this allows us to quantify the signal of the runout component, compared to a pure analysis of release susceptibility.

**Table 4:** Summary of computational experiments conducted in the present study

Experiment	Approach	CDF (A) or PDF (B)	Remarks
A1	Bottom-up (A)	Observed angle of reach *ω*_OT_	Conducted alternatively with spatially constant and spatially varied *P*_R_
A2	Bottom-up (A)	Observed travel distance *L*_OT_	
A3	Bottom-up (A)	Combination of *ω*_OT_ and *L*_OT_	
B1	Top-down (B)	Observed angle of reach *ω*_OT_	Conducted alternatively with spatially constant and spatially varied *LRSI*
B2	Top-down (B)	Observed travel distance *L*_OT_	
B3	Top-down (B)	Combination of *ω*_OT_ and *L*_OT_	

An issue of central importance consists in the strict separation of the data used for training of the model (in the present work, the derivation of the PDFs and CDFs) and the data used for the validation of the results. For each of the six experiments, the Schnepfau investigation area is divided into four subsets (W–Z in Fig. 4) in order to separate between a training area (TA) and a validation area (VA) (Table 2). The division lines between the subsets follow catchment boundaries in order to ensure that all landslides are clearly assigned to one of the four subsets and no landslide may impact more than one subset. Each computational experiment is repeated for four times, where three of the subsets are used as TA and one subset is used as VA. The resulting raster maps of *P*_L_ and *ILSI* are validated against the OIA by means of ROC plots. Thereby, only the VA is considered. The maps and the *AUROC* values derived for the four spatial subsets are averaged in order to derive the final result of each experiment. The relative level of conservativeness associated with each experiment is expressed by the averages of *P*_L_ and *ILSS* (*μ*_PL_ and *μ*_ILSS_, respectively). For the experiments A1–A3, the factor of conservativeness (*FOC*) is computed as the ratio between *μ*_PL_ and the fraction of OIA compared to the total size of the investigation area (De Lima Neves Seefelder et al. 2017). Values of *FOC* > 1 indicate an overestimation of the landslide susceptibility, compared to the observation, whereas values of *FOC* < 1 indicate an underestimation.

Building of the zonal statistics and the back-calculation of the PDFs and CDFs are conducted at a raster cell size of 5 m, whereas *P*_I_, *P*_L_, and *ILSS* are computed at a cell size of 10 m. The final results, however, are resampled to, and validated at, a cell size of 25 m which is in accordance with the cell size of the *LRSI* map. For back-calculating *ω*_OT_ and *L*_OT_, we start a set of 10^4^ random walks from each cell in the ORA of the TA. For computing *P*_I_, we start a set of 10^2^ random walks from each cell in the entire investigation area. We use Gaussian distributions to generate the PDFs and CDFs. The input parameters governing the behavior of the random walks consist of a weighting factor for the slope, a weighting factor and a control length for the perpetuation of the flow direction, the maximum run-up height, and the segment length for computing the travel distance. Their values are chosen in accordance with the suggestions of Mergili et al. (2015) who also describe these parameters in more detail.

## Results

### PDFs and CDFs of angle of reach and travel distance

**Fig. 5:**
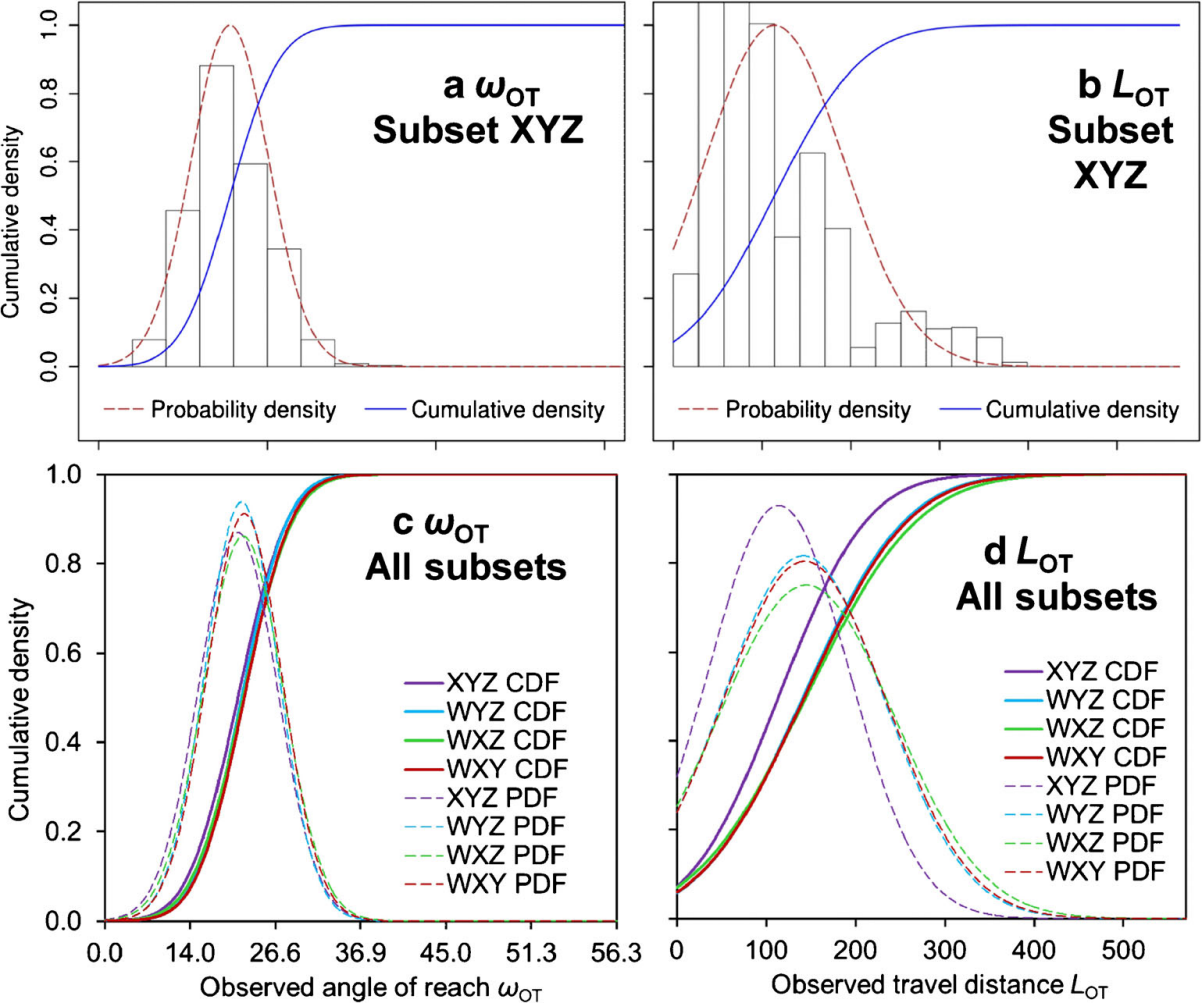
Gaussian probability density functions (PDFs) and cumulative density functions (CDFs) of the observed angle of reach *ω*_OT_ and the observed travel distance *L*_OT_ (Fig. 2). **a**, **b** Functions and histogram exemplified for the subset XYZ. **c**, **d** Overlay of the functions derived for the subsets XYZ, WYZ, WXZ, and WXY

Figure 5 illustrates the PDFs and CDFs of the observed angle of reach (*ω*_OT_) and the observed travel distance (*L*_OT_) (Fig. 2), thereby depicting the details derived for the subset XYZ applied as TA, and the variations observed among the results obtained for the various subsets. Whereas the Gaussian PDF for the angle of reach appropriately represents the histogram of the observed values (Fig. 5a), this is not the case for the travel distance, where the histogram indicates a bi- or even tri-modal distribution not following any obvious pattern (Fig. 5b). This observation underlines the deeper physical meaning of the angle of reach which, though complex in nature and influenced by various factors, serves as an approximation for the basal friction angle. The travel distance may be even more a complex function of several parameters such as the local terrain and the release volume.

The validation of *LRSI* against the OIA results in an AUROC value of 0.700. This value is taken as a reference for the evaluation of the added value of the combined approach (signal of the runout component of the model).

### Experiments A1–A3: bottom-up approach

**Fig. 6:**
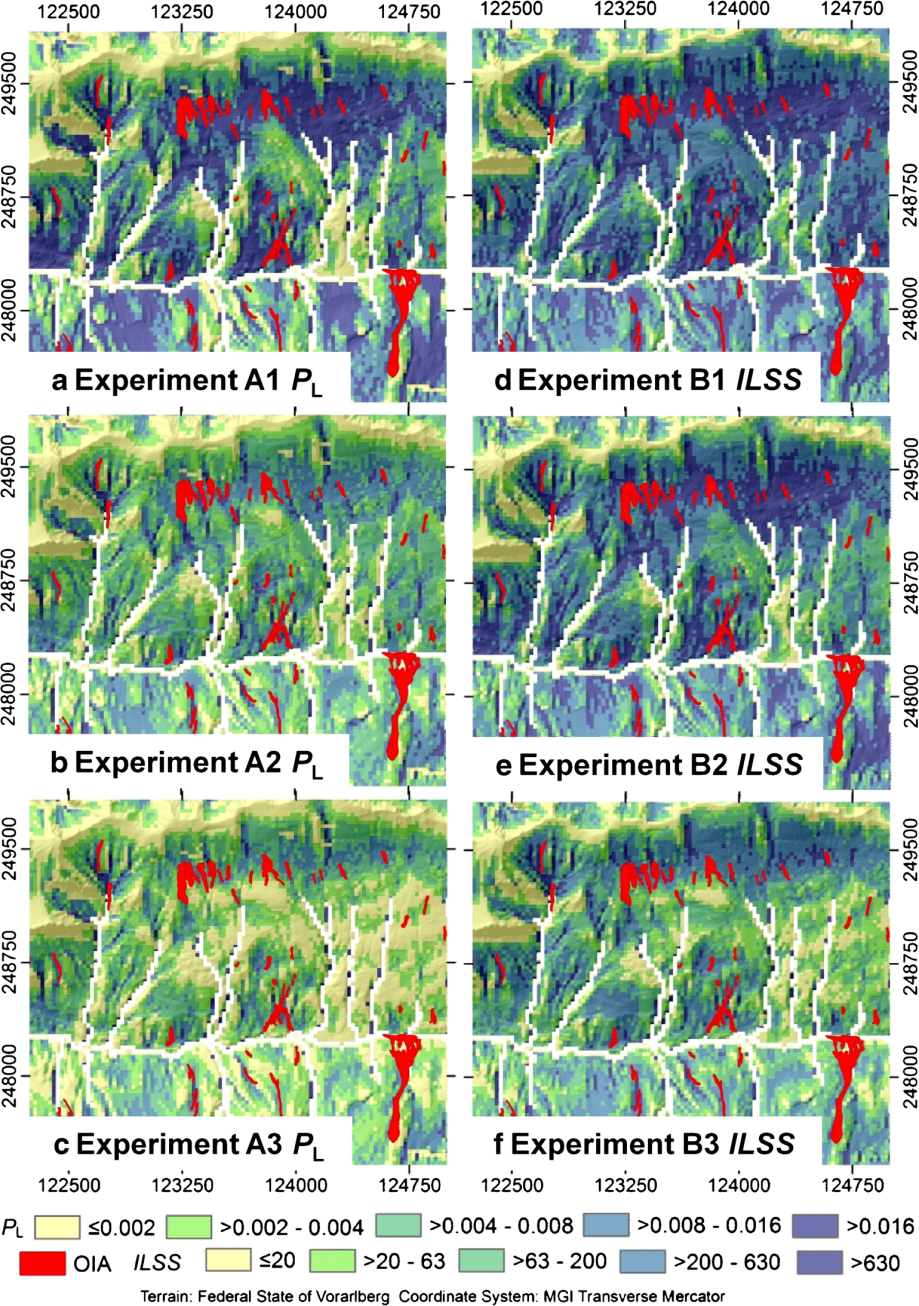
Maps of integrated landslide probabilities *P*_L_ (approach A) and integrated landslide susceptibility scores *ILSS* (approach B) derived for each of the computational experiments summarized in Table 3 (see Table 5 for the associated validation outcomes). For readability, only a small detail of the investigation area is shown (Fig. 4)

The integrated landslide probability *P*_L_ maps derived with the bottom-up approach are shown in Fig. 6a–c for a detail of the investigation area. Using the CDF of the observed angle of reach to constrain the runout distance (experiment A1) results in an *AUROC* value of 0.713, whereas *μ*_PL_ = 0.0130 (Table 5). This means that the integrated model can be classified as conservative (*FOC* = 1.75). The latter number is confirmed by the patterns shown in the *P*_L_ map: those areas with high values of *P*_L_ occupy most of the steeper slopes and reach down to the flow channels (Fig. 6a). The results become markedly less conservative when using the CDF of the travel distance (experiment A2): *μ*_PL_ = 0.0044 and *FOC* = 0.59. These numbers are confirmed by the patterns shown in Fig. 6b, where the areas with high values of *P*_L_ are reduced, compared to Fig. 6a. The model performance of A2 (*AUROC* = 0.724) is slightly better than in the experiment A1. Areas with high values of *P*_L_ are even more reduced in the experiment A3: the lower level of conservativeness (*μ*_PL_ = 0.00306 and *FOC* = 0.41) is explained by the multiplication of the two CDFs. The model performance of A3 (*AUROC* = 0.637) is much worse than it is for the experiments A1 and A2. The lower performance associated with the experiment A3 is related to low values of *P*_L_ near to the termini of some of the observed transit and deposition areas (Fig. 6c). The experiments A1 and A2 result in an only slightly higher performance in terms of *AUROC* in comparison to an analysis of the release susceptibility only, whereas the approach A3 even results in a clearly worse performance.

**Table 5:** Key indicators describing the performance and conservativeness of the computational experiments introduced in Table 3. Note that the results obtained with spatially uniform values of *P*_R_ or *LRSI* only serve as a reference for the evaluation of the signal of the release component of the model. *NA* not available

Experiment	With spatially varied *P*_R_ or *LRSI*				With spatially uniform *P*_R_ or *LRSI*			
	*AUROC*	*μ*_PL_	*FOC*	*μ*_ILSS_	*AUROC*	*μ*_PL_	*FOC*	*μ*_ILSS_
A1	0.713	0.0130	1.75	NA	0.738	0.0139	1.87	NA
A2	0.724	0.00440	0.59	NA	0.748	0.00485	0.65	NA
A3	0.637	0.00306	0.41	NA	0.664	0.00347	0.47	NA
B1	0.774	NA	NA	411	0.700	NA	NA	517
B2	0.824	NA	NA	394	0.733	NA	NA	431
B3	0.727	NA	NA	129	0.593	NA	NA	162

**Fig. 7:**
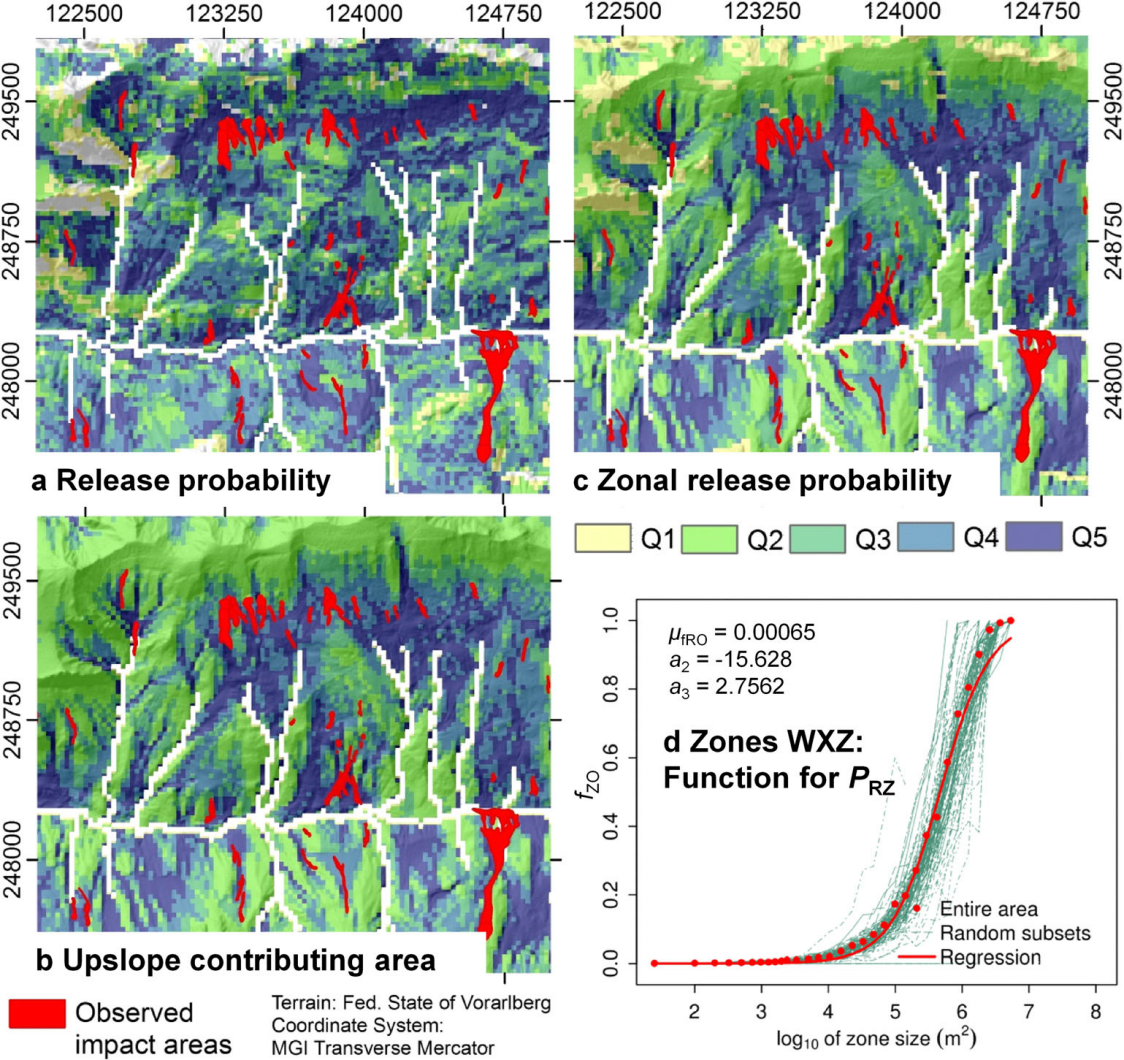
Set of results of the computational experiment A1. For readability, only a small detail of the investigation area is shown in **a**–**c** (Fig. 4). Each map is classified according to the quintiles Q1–Q5 of its range of values. **a** Release probability *P*_R_. **b** Upslope contributing area *Z* (km^2^) related to each raster cell. **c** Zonal release probability *P*_RZ_. **d** Function for the zonal release probability *f*_ZO_, exemplified for the subset WXZ. *P*_RZ_ is derived by scaling this function to the average of *P*_R_ within the upslope contributing area of the considered cell (Appendix Fig. 9)

Further, the results of the experiments A1–A3 are not very sensitive to whether the spatial differentiation of *P*_R_ is considered or not, in terms of both *AUROC* and *FOC* (Table 5). Most interestingly, *AUROC* is even by approx. 0.025 higher when assuming a spatially uniform value of *P*_R_. These observations are clearly a result of smoothing associated with the zonal release probability, as confirmed by Fig. 7: there is a strong visual correspondence between the size of the upslope contributing area (Fig. 7b) and *P*_RZ_ (Fig. 7c), whereas no visual similarity is observed between *P*_R_ (Fig. 7a) and *P*_RZ_. Figure 7d illustrates the function used for the deduction of *P*_RZ_ derived from the inventory of the ORA (Appendix Fig. 9), exemplified for the subset WXZ used as TA.

### Experiments B1–B3: top-down approach

The integrated landslide susceptibility index *ILSS* maps derived with the top-down approach are shown in Fig. 6d–f, whereas the associated performance and conservativeness indicators are summarized in Table 5. As the absolute numbers of *ILSS* are meaningless, quantiles are shown in the legends of Fig. 6d–f. Further, *FOC* cannot be derived with the approach B, so that we compare the averages of *ILSS*, *μ*_ILSS_, in order to learn about the differences in conservativeness among the results of the computational experiments. We now analyze the spatial patterns of *ILSS* and the associated model performance and conservativeness in the light of comparison with the results for the bottom-up approach. As the most general observations we note that:

- The visual patterns of each *ILSS* map are largely similar to the *P*_L_ map derived with the corresponding experiment of approach A. Values of *μ*_ILSS_ decrease from 411 to 394, and then drastically to 129, from experiments B1–B3.
- B2 clearly shows the best model performance (*AUROC* = 0.824) among the experiments B1–B3, whereas *AUROC* = 0.774 for B1 and *AUROC* = 0.727 for B3. This means that the general trend largely corresponds to the trends observed among the experiments A1–A3, with the more pronounced optimum obtained with the experiment B2 (Table 5).
- The model performance associated with *ILSS* is always higher than the performance associated with the corresponding *P*_L_ maps.

There are two further important differences among the results obtained with the approaches A and B. First, the general performance in terms of *AUROC* is clearly higher than the performance of a pure analysis of release susceptibility in approach B. This is particularly true for the experiments B1 (difference of 0.074) and B2 (difference of 0.124).

Second, in approach B, the model performance in terms of *AUROC* is clearly weaker when using spatially uniform values of *LRSI* instead of the spatially varied maps (differences of 0.074, 0.091, and 0.134 for the experiments 1, 2, and 3). The corresponding experiments with the approach A are less sensitive to the *P*_R_ map (Table 5). This discrepancy indicates that the signal of the release component makes it all the way to the final result in approach B. This is not the case in approach A, where the noise even leads to slightly higher *AUROC* values with constant *P*_R_. This may also explain the generally higher *AUROC* values obtained in the experiments B1–B3, compared to A1–A3: the signal of *LRSI* most likely improves the model performance in terms of empirical adequacy.

## Discussion

We have presented and compared two innovative approaches to approximate the susceptibility of any point in a landscape—represented by a GIS raster cell—to be affected by shallow landslide processes or the resulting hillslope debris flows, be it through release or through runout.

Approach A employs a bottom-up method and, from a technical point of view, yields a “true” integrated landslide probability *P*_L_, whereas the approach B uses a more intuitive and straightforward top-down method in order to yield a semi-quantitative landslide susceptibility score *ILSS*. Within the bottom-up approach, the concept of the zonal release probability *P*_RZ_ is employed. The purpose of *P*_RZ_ consists in the appropriate consideration of the probability of landslide release within the upslope contributing area of a given cell (Fig. 3, Appendix Fig. 9). This is necessary to make the result a “true” probability, where *FOC* ≈ 1, but is done at the cost of smoothing of the spatial patterns of the cell-based release probability *P*_R_. No such smoothing is required within the approach B. As a consequence, the signal of *P*_R_ does not survive the routing procedure in the approach A, whereas it does in the approach B (Table 5, Fig. 6). Model performance in terms of empirical adequacy is therefore better in approach B. We conclude that approach B is certainly most suitable in cases where a semi-quantitative spatial overview of landslide susceptibility is required, whereas approach A is better suitable in cases where “true” probabilities are needed, e.g., as part of quantitative risk analyses. However, in doing so, it also has to be kept in mind that even comprehensive landslide inventories are never complete but just represent a snapshot, e.g., depicting only those landslides associated with one or more rainstorm events. Converting spatial probabilities in spatio-temporal probabilities therefore remains a challenge.

**Fig. 8:**
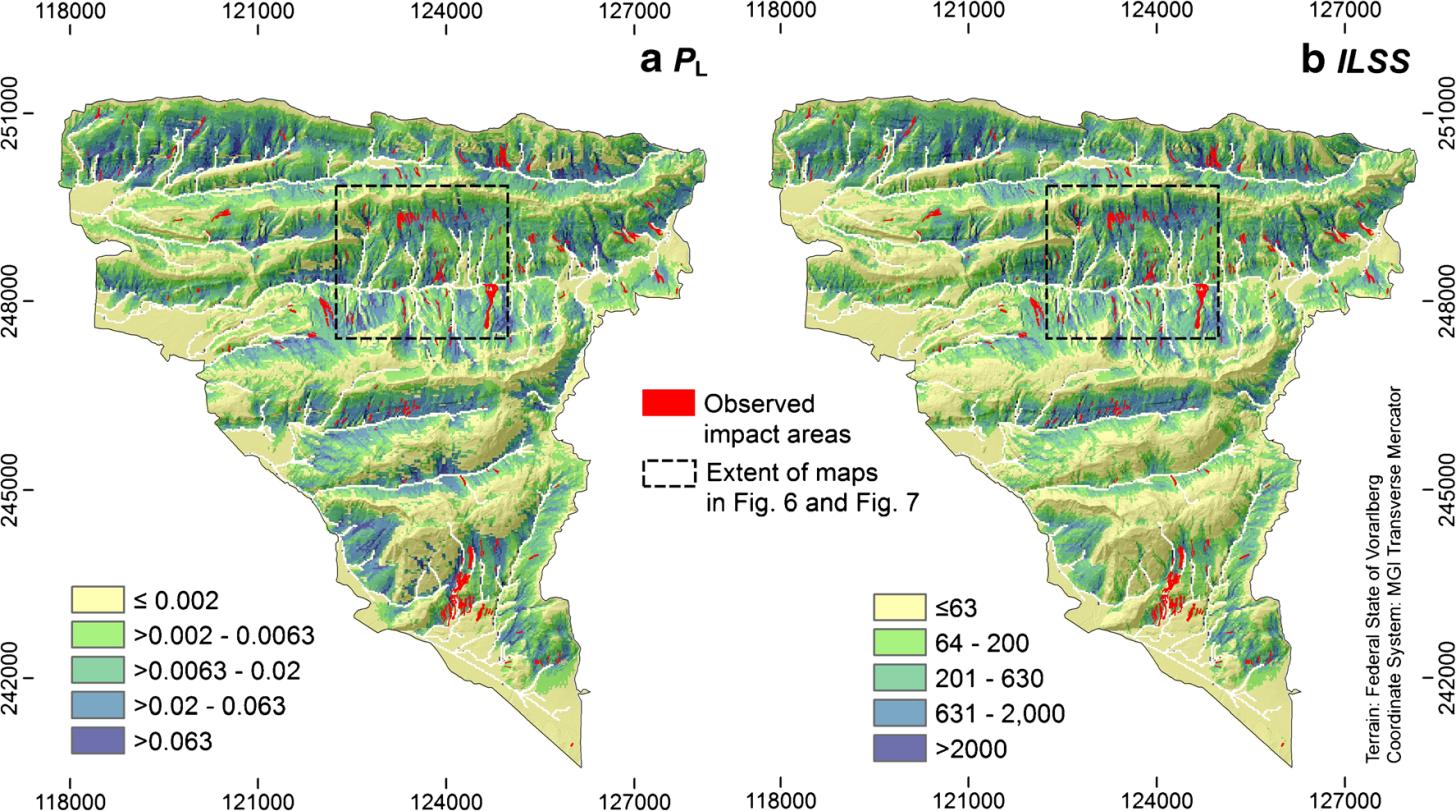
(a, b) Maps of integrated landslide probability *P*_L_ and integrated landslide susceptibility score *ILSS* for the entire investigation area, derived with the computational experiments A1 and B1, respectively. Considering the validation outcomes summarized in Table 5 and their interpretation, we recommend these results for further use

The model results generally show a fair to (mostly) good performance when compared to the observed impact areas (Table 5). However, performance does not only vary as a response to the choice of the approach, but also to the CDF or PDF employed. The physically most meaningful parameter is the angle of reach, serving as a rough approximation of the basal friction angle: in the present work, its application results in the best model performance in terms of *AUROC*, but also in very conservative results. The latter observation is related to the fact that the investigation area is characterized by long slopes of uniform inclination, leading to high impact probabilities even far below terminal points of the observed events. The results are high values of *FOC* in the experiments A1 and B1. Application of the travel distance is physically less meaningful, but not susceptible to factors such as uniform slope inclination. The results of the experiments A2 and B2 are therefore less conservative. Particularly in the experiment A2, *P*_L_ is clearly underestimated, compared to the observation, whereas the difference between B1 and B2 is minor. However, these experiments show higher *AUROC* values than their counterparts A1 and B1, respectively. Combining angle of reach and travel distance results in the lowest performance and conservativeness—here, the lower parts of some observed transit and deposition areas coincide with areas of low *P*_L_ or *ILSS* (experiments A3 and B3). In summary, we consider the results obtained with the density functions of the angle of reach most appropriate. The corresponding *P*_L_ and *ILSS* maps for the entire investigation area are presented in Fig. 8.

Whereas traditional statistically based landslide susceptibility studies (e.g., Carrara et al. 1991; Baeza and Corominas 2001; Dai et al. 2001; Lee and Min 2001; Saha et al. 2005; Guzzetti 2006; Komac 2006; Lee and Sambath 2006; Lee and Pradhan 2007; Yalcin 2008; Yilmaz 2009; Nandi and Shakoor 2010; Yalcin et al. 2011; Schwarz et al. 2009; Tilch et al. 2011; De Graff et al. 2012; Petschko et al. 2014; Schwarz and Tilch 2017, 2018) are useful to identify likely release areas at the level of GIS raster cells, their results to play a limited role when considering integrated landslide probability (approach A). This is—as it was already discussed—most probably a result of the strong correlation between zone size and *P*_RZ_—and, consequently, the non-existent reflection of *P*_R_ in *P*_L_ (Fig. 7). However, the results summarized in Table 5 reveal a decreasing signal of *LRSI* with increasing travel distance in approach B, where no smoothing is applied. The difference in *AUROC* between the spatially varied and the spatially uniform input of *LRSI* is smallest in the experiment B1 (longest travel distances due to the effects discussed above) and largest in the experiment B3 (shortest travel distance). Hence, the longer the travel distance becomes, the more dominant are the effects of propagation, as the signal of *LRSI* gets lost in superimposing random walks originating from many release cells. The poor signal of the release component in approach A leads to a performance level comparable to a pure release susceptibility analysis, where runout is neglected. Only in approach B, where the signals of release and runout are both reflected in the results, the *AUROC* values clearly exceed a “base level” of approx. 0.7.

As a consequence of what was said above, the suggested methods are considered particularly useful for those situations where landslides are highly mobile, e.g., where shallow landslides convert into hillslope debris flows, as it is reflected in the inventory used for the present study. The methods have to be used with care where landslides are less mobile. In these cases, the PDF and CDF of the angle of reach or of the travel distance would reflect the length distribution of the observed release areas rather than the mobility of the landslides. In general, we note that the angles of reach used in the present study rely on another concept than those included in published relationships (e.g., Scheidegger 1973; Zimmermann et al. 1997; Rickenmann 1999; Corominas et al. 2003; Noetzli et al. 2006). Whereas these and other authors refer to the angle between the highest and the terminal point of the landslide, we consider the angles between any release cell of an observed or hypothetic landslide and its terminal point (Fig. 2). This applies to the travel distance in an analogous way and is required as the random walks are started from all raster cells, which may coincide with any random position within landslide release areas, but not necessarily with the highest point of a landslide. Further, it is not possible to make runout dependent on landslide volumes in a straightforward way as it was done, e.g., by Scheidegger (1973), Rickenmann (1999), or Noetzli et al. (2006). Such approaches are useful for single events with known volumes. As the volumes of possible future landslides are not a priori known at the scale relevant for the present study, we rely on the plain PDFs and CDFs.

The runout is modeled only for hillslope debris flows, whereas flow channels have to be excluded from all the analyses. At such a slopechannel interface, we have no empirical evidence for the change of landslide behavior. For instance, landslides may stop due to the sudden drop in slope angle, or the material deposited may be mobilized by flowing water. This means that the methods, as applied in the present study, are not suitable to predict the possible impacts of extreme events and process chains at the outlet of flow channels. More data and some adaptations would be necessary to do so: a future direction could be to include the conditions along the landslide path (distinction between slopes and channels, type of basal material, vegetation) in the parameterization of the random walks.

## Conclusions

We have presented and compared two approaches for integrated statistical modeling of landslide susceptibility at catchment or even broader scales. These methods were applied to shallow landslides developing into hillslope debris flows in the Schnepfau investigation area in western Austria. A map showing the spatial patterns of the landslide release susceptibility index was used as the basis. Approach A—characterized as bottom-up approach—employs the concept of the zonal release probability (Mergili and Chu 2015), approximating the probability that landslide release occurs from at least one place in the upslope contributing area of a given raster cell, whereas the approach B—characterized as top-down approach—works directly with the cell-based release susceptibility index and is therefore more straightforward and intuitive at the cost of providing only semi-quantitative results. Both methods impose a constrained random walk approach onto the release in order to compute landslide propagation (Mergili et al. 2015) and thereby rely on the density functions of the angles of reach and/or travel distances of the observed landslides.

The final outcome approximates the quantitative spatial probability (approach A) and the semi-quantitative susceptibility (approach B) of a GIS raster cell to be affected by a landslide either through its release or through its propagation. Analyzing the results of the procedure leads us to a set of key conclusions:

- Most computational experiments have performed well or fair in terms of empirical adequacy (*AUROC*), possibly indicating a certain degree of validity of both approaches. The visual appearance of the final maps is largely similar among the two approaches. The approach B generally performs better than the approach A. This observation is most likely associated with the better representation of the release susceptibility in the approach B, whereas the release probability is smoothed out in the approach A, so that the result is dominated by landslide propagation. We therefore recommend using the approach B for semi-quantitative spatial overviews, whereas the approach A is still needed as the basis of quantitative risk analyses. We conclude that there is a profit of combining release and propagation models. This profit is clearly reflected in those *AUROC* values obtained with the approach B (Table 5). However, we still need a better way to consider the release probability in a fully quantitative way without losing too much of its signal (approach A).
- We further conclude that employing the travel distance as break criterion yields the best overall performance particularly in approach B, even though the angle of reach appears most suitable from a physical point of view. Using a combination of angle of reach and travel distance leads to an underestimate of landslide susceptibility. However, the influence of the type of probability functions used remains a question of future research.
- In approach B, the signal of the spatial differentiation of the release component weakens with increasing travel distance. Whereas the results of the integrated analyses are particularly useful for situations with highly mobile events, the propagation of landslides through flow channels as channelized debris flows is excluded due to lacking empirical evidence and missing computational implementation. This, besides the loss of the signal of *P*_R_ with approach A, remains one of the major limitations of the approaches and means that analyzing the possible impact of extreme events and complex process chains is out of scope of the present study.

As a consequence thereof, future enhancements could go in the direction of including the conditions along the landslide path in the parameterization of the random walks, in order to be able to consider also more complex situations at the small catchment scale. The spatial diversity of geological or if possible rheological conditions should be included into the modeling process.

## Figures and Tables

**Fig. 9:**
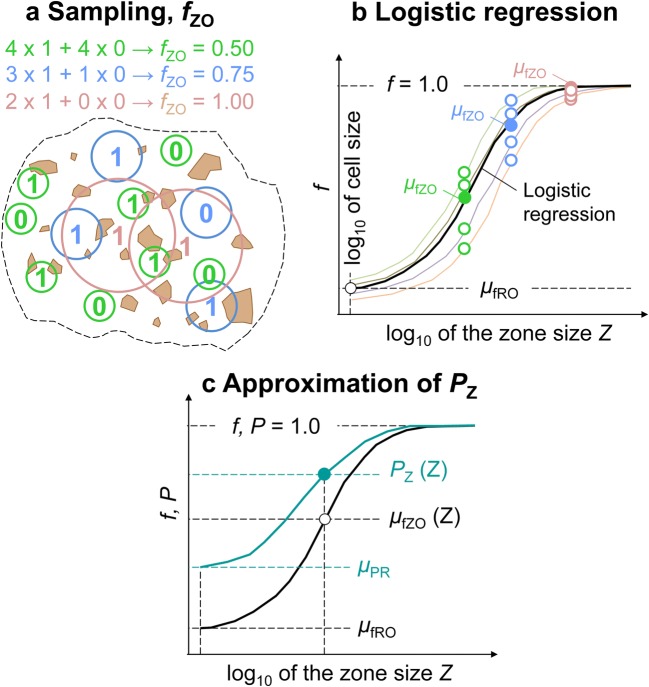
Approximation of the zonal release probability *P*_RZ_ (approach A). **a** Sampling of subsets of the test areas in order to estimate *f*_ZO_ associated with a given range of zone size *Z*. **b** Line cloud of *f*_ZO_ (*Z*). The logistic regression is derived from the average values *μ*_fZO_ of *f*_ZO_ for the sampled values of *Z*. **c** Approximation of *P*_RZ_ (*Z*) for a given value of *μ*_PR_, assuming that the shape of the curve is insensitive to *μ*_PR_

## Appendix

We now describe in detail the empirical approximation procedure used to derive the zonal release probability *P*_RZ_ (Appendix Fig. 9):

1. A subset of the TA with random size and random center coordinates is selected (“test area”). *f*_RO_ is the fraction of observed landslide release cells in the test area (i.e., the fraction of ORA cells out of all data cells).
2. Within the test area, a set of subsets with constant zone size *Z* and randomized center coordinates is evaluated for the presence of observed landslide release cells. *f*_ZO_ is defined as the fraction of subsets with at least one observed landslide release cell (Appendix Fig. 9a).
3. Step 2 is repeated for a large number of sets of subsets covering a broad range of values of *Z*.

The steps 1–3 are repeated for a large number of test areas.

This procedure results in a line cloud of *f*_ZO_ plotted against *Z* (one line for each subset; Appendix Fig. 9b). A logistic regression is fitted to the average value of *f*_ZO_, *μ*_fZO_, for each tested value of *Z*:

2 $$ {\mu}_{\mathrm{fZO}}(Z)=\frac{1-{\mu}_{\mathrm{fRO}}}{1+{e}^{-\left({a}_2+{a}_3Z\right)}}+{\mu}_{\mathrm{fRO}} $$

*a*_2_ and *a*_3_ are the regression coefficients, whereas *μ*_fRO_ is the average of *f*_RO_ over all test areas. *μ*_fZO_ (*Z*) and *μ*_fRO_ are the basis for the computation of *P*_RZ_ (Eq. 3). We emphasize that *μ*_fZO_ (*Z*) is purely derived from the “density” of observed landslides, assuming a uniform landslide release probability over the entire training area. However, if the requirement that the average of the cell-based landslide release probability *P*_R_ over the entire TA, *μ*_PRG_, has to be equal to the landslide density in the TA is met, then *μ*_PRG_ ≈ *μ*_fRO_.

In order to predict the zonal release probability with regard to the upslope contributing area of size *Z* (m^2^) of a given cell, we have to assume that the shape of the logistic regression function is insensitive to the average of all cell-based release probabilities *μ*_PR_ in the upslope contribution area (Mergili and Chu 2015; Appendix Fig. 9c): the zonal release probability *P*_RZ_ (Z) is then computed as.

3 $$ {P}_{\mathrm{RZ}}(Z)\approx 1-\left(1-{\mu}_{\mathrm{fZO}}(Z)\right)\frac{1-{\mu}_{\mathrm{PR}}}{1-{\mu}_{\mathrm{fRO}}} $$
